# Time-delay signature suppression in delayed-feedback semiconductor lasers as a paradigm for feedback control in complex physiological networks

**DOI:** 10.3389/fnetp.2023.1330375

**Published:** 2024-01-11

**Authors:** Yanhua Hong, Zhuqiang Zhong, K. Alan Shore

**Affiliations:** ^1^ School of Computer Science and Engineering, Bangor University, Bangor, United Kingdom; ^2^ College of Science, Chongqing University of Technology, Chongqing, China

**Keywords:** chaos, network physiology, feedback control, nonlinear dynamics, semiconductor lasers

## Abstract

Physiological networks, as observed in the human organism, involve multi-component systems with feedback loops that contribute to self-regulation. Physiological phenomena accompanied by time-delay effects may lead to oscillatory and even chaotic dynamics in their behaviors. Analogous dynamics are found in semiconductor lasers subjected to delayed optical feedback, where the dynamics typically include a time-delay signature. In many applications of semiconductor lasers, the suppression of the time-delay signature is essential, and hence several approaches have been adopted for that purpose. In this paper, experimental results are presented wherein photonic filters utilized in order to suppress time-delay signatures in semiconductor lasers subjected to delayed optical feedback effects. Two types of semiconductor lasers are used: discrete-mode semiconductor lasers and vertical-cavity surface-emitting lasers (VCSELs). It is shown that with the use of photonic filters, a complete suppression of the time-delay signature may be affected in discrete-mode semiconductor lasers, but a remnant of the signature persists in VCSELs. These results contribute to the broader understanding of time-delay effects in complex systems. The exploration of photonic filters as a means to suppress time-delay signatures opens avenues for potential applications in diverse fields, extending the interdisciplinary nature of this study.

## 1 Introduction

In the human organism, multi-component physiological systems, each with its own regulatory mechanism, continuously interact to coordinate their functions in an integrated network (Ivanov 2021). This leads to complex nonlinear dynamics, and many examples of such self-organized pattern formation in physiological networks have been elucidated (Berner et al., 2022; Sawicki et al., 2022). Lasers have been used as a paradigm of complex nonlinear dynamics occurring in a wide variety of much more complex biological and physiological systems since the pioneering work by Graham and Haken (1970) and Haken (1975) and in particular, for time-delayed feedback control, refer to Schöll et al. (2010). In physiological networks, feedback loops act simultaneously in self-regulated physiological systems (Healy et al., 2021; More et al., 2023).

The study of time-delay effects has been identified as an aid to characterizing physiological systems and their regulatory mechanisms. It is found, for example, that oscillations and chaos can be established in blood flow due to time-delay effects (Holstein-Rathlou, 1993). Analogous oscillatory and chaotic behaviors have been studied in considerable theoretical and experimental detail in semiconductor lasers subjected to delayed optical feedback (Soriano et al., 2013). Because of their ease of operation, semiconductor lasers offer a convenient testbed for exploring the diverse dynamical behavior which may arise when the laser is subjected to optical feedback (Kane and Alan Shore, 2005; Ohtsubo, 2013). There is a considerable variety of semiconductor lasers, and their response to such time-delayed optical feedback is dependent on the detailed characteristics of the lasers. In turn, such varieties of behaviors may be instructive for the exploration of dynamical behaviors arising in physiological systems in which time-delay effects play a significant role in determining physiological phenomena.

In general, when time delays are the drivers of dynamics, there is a characteristic signature of those delays contained within the system dynamics. The finite time of signal propagation between nodes of a network may manifest itself as a time-delay signature. Such a signature is often undesirable, and hence effort has been made to suppress the time-delay signature. Thus, for example, in the case of chaotic semiconductor lasers being used for secure communications (Argyris et al., 2005), the persistence of a time-delay signature may compromise the security of data transmission (Rontani et al., 2009). In this context, substantial efforts have been dedicated to erase time-delay signatures (Shahverdiev and Shore, 2009; Nguimdo et al., 2011; Li et al., 2012; Li et al., 2012; Hong, 2013; Wang et al., 2013; Zhong et al., 2013; Hong, Spencer, and Shore, 2014; Xiang et al., 2014; Li and Chan, 2015; Hong et al., 2016; Mu et al., 2016; Wang et al., 2017; Zhang et al., 2017; Jiang et al., 2018; Li et al., 2018; Zhang et al., 2018; Zhao et al., 2019; Ma et al., 2020; Zhang et al., 2020; Cui et al., 2022). These efforts encompass various methods, including modulated optoelectronic feedback, distributed feedback from a fiber Bragg grating, phase-modulated feedback, chaos optical injection, mutual injection, and cascaded injection, and the influence of factors, like fiber scattering and dispersion. Most of these investigations have focused on vertical-cavity surface-emitting lasers (VCSELs) or distributed feedback (DFB) semiconductor lasers.

However, recent research has uncovered the unique characteristics of chaos generated in discrete-mode (DM) semiconductor lasers, demonstrating the possibility of achieving flat broadband chaos through optical feedback under optimized conditions (Chang et al., 2020). However, the study of the time-delay signature of chaos generated in optically injected DM lasers remains unexplored. DM lasers are a distinct type of Fabry–Pérot (FP) lasers that etch a small number of features along the ridge waveguide, modifying the cavity spectrum to amplify a single cavity mode while suppressing the others, ensuring single-mode operation (Osborne et al., 2007). DM lasers offer several advantages, including cost-effectiveness, resilience to optical feedback, stable single-mode emission, a broad operational temperature range, and high bandwidth. In this paper, a novel approach to eliminating time-delay signatures using photonic filters in a DM laser is explored. To facilitate comparison, the same experimental configuration is applied to a VCSEL. The findings of this study underscore the exceptional efficacy of photonic filters in suppressing time-delay signatures in DM lasers, whereas in the case of VCSELs, complete signature suppression remains elusive.

## 2 Experimental setup

**FIGURE 1:**
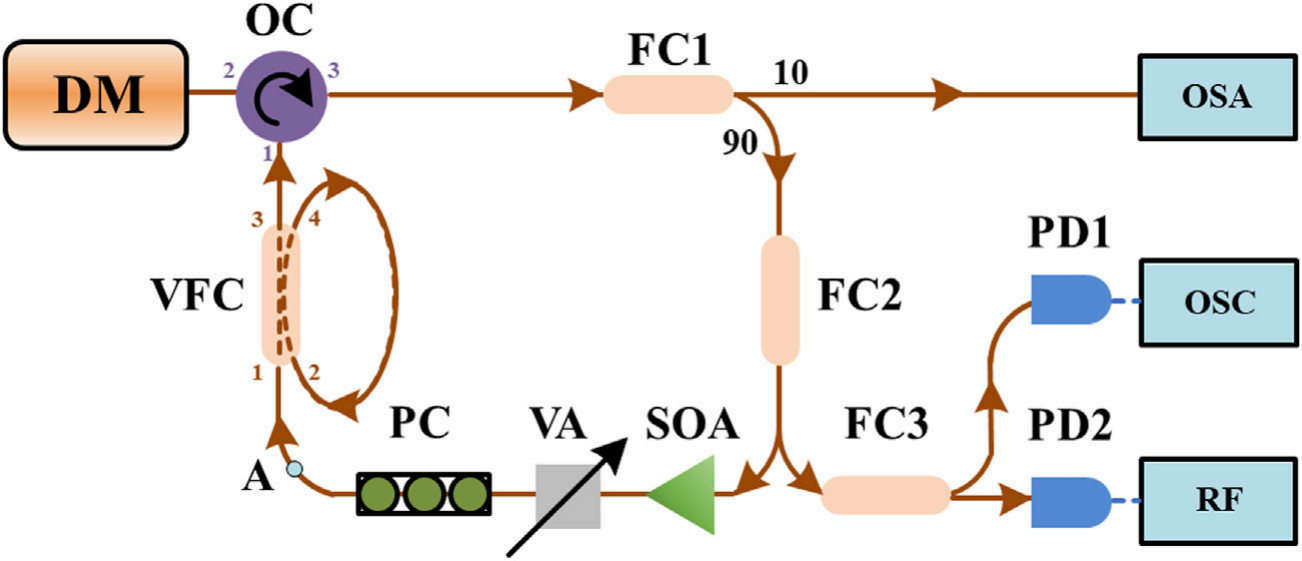
Experimental setup.

The schematic experimental setup is shown in Figure 1. In this experiment, two distinct types of laser diodes (LDs) are employed. First, we utilize a DM laser (EP1550-DM-01-FA) from Eblana Photonics. Second, we employ VCSELs of RayCan RC330001-FFA type. Both LDs are driven by a low-noise current source (Thorlabs LDC201 CU) and maintained at room temperature by a highly precise temperature controller (Lightwave LDT-5412), with a lasing wavelength of approximately 1,550 nm.

For the conventional feedback setup, the feedback loop is formed by an optical circulator (OC), fiber couplers (FC1 and FC2), a semiconductor optical amplifier (SOA), a variable optical attenuator (VA), and a polarization controller (PC). Within this feedback loop, SOA serves to amplify the feedback power, VA is used to adjust the feedback power, and PC regulates the polarization of the feedback beam to ensure maximum efficiency on the dynamics of the lasers.

In the photonic filter feedback setup, a variable fiber coupler (VFC: Newport F-CPL-1550_N-FA FC3) is integrated into the feedback loop, as indicated by the dashed frame. The photonic filter feedback configuration is established by connecting ports 2 and 4 of the VFC. This arrangement is commonly referred to as an infinite impulse response single-source microwave photonic filters (IIR SSMPFs) or fiber ring resonators, and its specification details have been comprehensively discussed in Capmany, Ortega, and Pastor (2006).

In the detection section, 10% of the optical power is split using FC1 and directed toward an optical spectrum analyzer (OSA: Agilent 86141B with a resolution of 0.06 nm) for optical spectrum measurements. Simultaneously, FC2 separates 50% of the power from the feedback loop and directs it to a third fiber coupler (FC3). FC3 further divides the optical power evenly and routes it to two photodetectors: a 12 GHz photodetector (PD1: New Focus 1544-B) and a 40 GHz photodetector (PD2: Thorlabs, RXM40AF). The outputs of PD1 and PD2 are recorded using an oscilloscope (OSC, Tektronix TDS7404) with a bandwidth of 4 GHz and an electrical spectrum analyzer (RF, R&S FSE-K20) with a bandwidth of 40 GHz, respectively. The oscilloscope operates at a sampling rate of 20 GS/s, with a total time duration of 2 µs.

In this paper, the optical feedback ratio is defined as the ratio of the feedback power to the output power of the free-running laser. The optical feedback power is the power of the feedback beam before it enters the laser. It is measured at port 1 of OC, taking into consideration the loss from port 1 to port 2 of the OC. In this experiment, we also investigate the effect of the coupling ratio of the VFC on the time-delay signature. The coupling ratio is defined as the percentage of the power transferred from port 1 to port 4 in the VFC, as shown in Figure 1.

## 3 Time-delay signature analysis methods

Numerous techniques are available for a qualitative assessment of the time-delay signature, such as mutual information (Rontani et al., 2009; Nguimdo et al., 2011; Li et al., 2012; Li and Chan, 2015; Wang et al., 2017; Zhang et al., 2017), autocorrelation coefficient (ACC) (Rontani et al., 2009; Shahverdiev and Shore, 2009; Li et al., 2012; Li et al., 2012; Hong, 2013; Wang et al., 2013; Zhong et al., 2013; Hong, Spencer, and Shore, 2014; Xiang et al., 2014; Li and Chan, 2015; Hong et al., 2016; Mu et al., 2016; Wang et al., 2017; Zhang et al., 2017; 2018; Jiang et al., 2018; Li et al., 2018; Zhao et al., 2019; Ma et al., 2020; Zhang et al., 2020; Cui et al., 2022), and permutation entropy (PE) (Hong, 2013; Zhong et al., 2013; Xiang et al., 2014; Mu et al., 2016; Cui et al., 2022). In this study, we utilize both ACC and PE methods to detect the time-delay signature. The ACC, denoted as C, is defined as follows:

$$C\left(\Delta t\right)=\frac{\langle \left[I\left(t+\Delta t\right)-\langle I\left(t+\Delta t\right)\rangle \right]\left[I\left(t\right)-\langle I\left(t\right)\rangle \right]\rangle }{\sqrt{\langle {\left[I\left(t+\Delta t\right)-\langle I\left(t+\Delta t\right)\rangle \right]}^{2}\rangle \langle {\left[I\left(t\right)-\langle I\left(t\right)\rangle \right]}^{2}\rangle }},$$

where *I* represents the output intensity of the laser, <⋅> denotes a time average, and Δt is the delay time. The value of *C* falls within the range of −1 to 1. A value of 1 signifies a complete positive correlation, while −1 indicates a full negative (anti) correlation. When the value is 0, it denotes a state of complete randomness, indicating no correlation whatsoever.

The PE method, initially introduced by Bandt and Pompe (2002), involves a time series {I_t_, t = 1, 2, … , N}, which represents the measures of the N samples of the output intensities of the laser. Given the time series {I_t_, t = 1,2, … , N}, subsets S_q_, each containing M samples (M > 1) of the measured intensities, are formed with an embedding delay time τ = nT_s_, where n is an integer number and T_s_ is the reciprocal of the sampling rate. The ordinal patterns of subsets are expressed as S_q_ = [I(t), I (t+τ), …I (t+(M-1)τ)]. For practical purposes, Bandt and Pompe recommended choosing M within the range of 3–7. In this work, we have selected M to be 5. Each subset S_q_ can be organized as [I (t+(r_1_−1)τ)≤I (t+(r_2_−1)τ)≤…≤I (t+(r_M_−1)τ)]. Thus, each subset can be uniquely represented as an “ordinal pattern” π = (r_1_, r_2_, … , r_M_), which is one of the possible permutations of subset S_q_ with M dimensions. The permutation entropy is derived from the probability distribution p(π) as follows:

$$p\left(\pi \right)=\frac{\#\left\{t|t\leq N-M-n+1;S_{q}hastype\pi \right\}}{N-M-n+1},$$

where the symbol # denotes “number.” The permutation entropy is then determined using the probability p(π) as follows:

$$h\left(p\right)=-\sum p\left(\pi \right)\log p\left(\pi \right).$$

## 4 Results

### 4.1 Discrete-mode laser

The DM laser used in this experiment has a threshold current of 12.5 mA at room temperature and is biased at 80 mA. Initially, conventional optical feedback is introduced by disconnecting ports 2 and 4 of the VFC.

**FIGURE 2:**
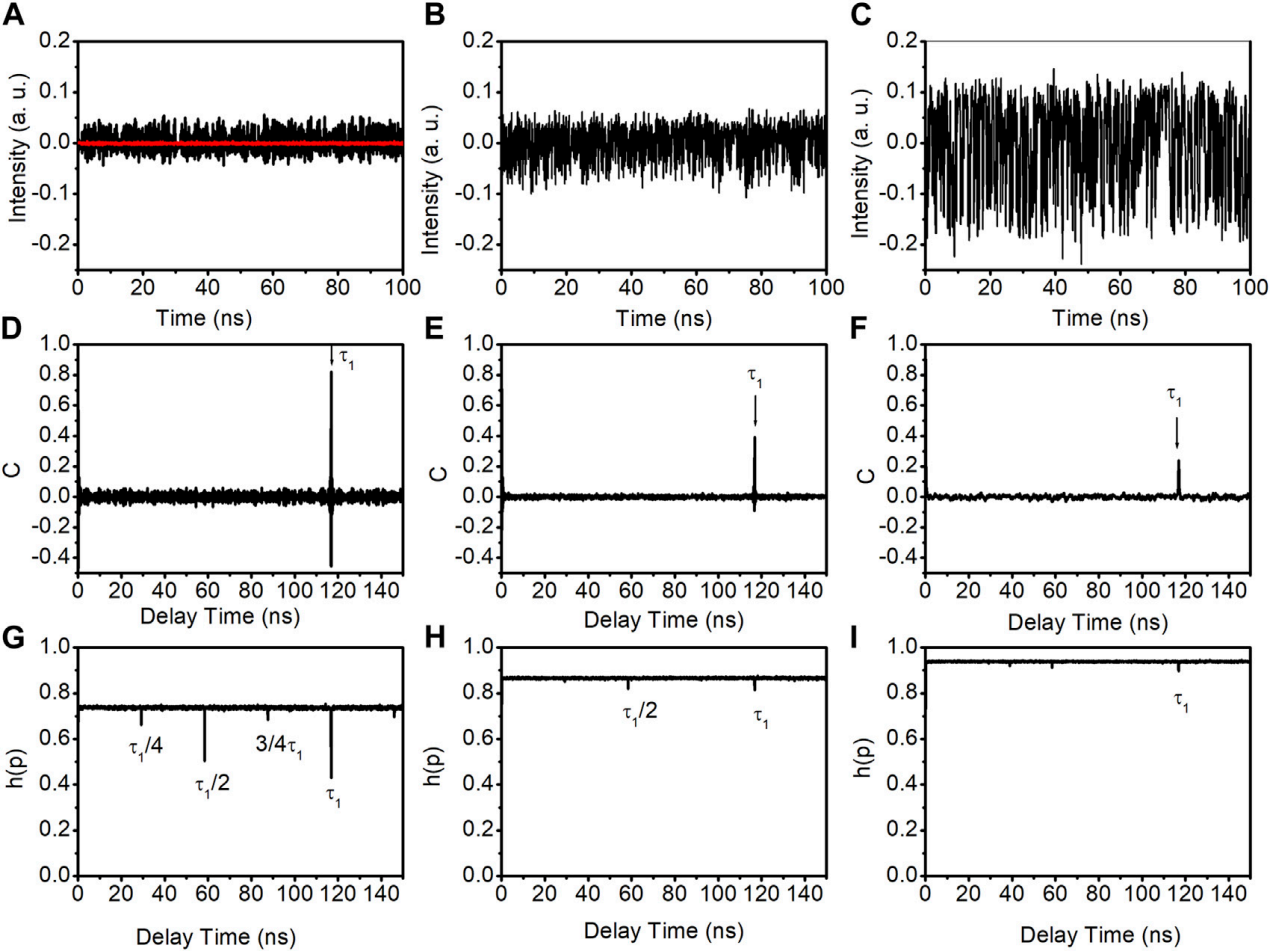
Time traces, ACC curves and PE curves of the output of the DM laser subjected to optical feedback. **(A–C)** are time traces with the feedback ratios of −14.5 dB, −10.5 dB, and −1.5 dB, respectively. **(D–F)** are autocorrelation coefficient curves with the feedback ratios of −14.5 dB, −10.5 dB, and −1.5 dB, respectively. **(G–I)** are PE curves with the feedback ratios of −14.5 dB, −10.5 dB, and −1.5 dB, respectively. The red line in **(A)** is the free-running DM laser output.

Figure 2 shows the time traces (top row), autocorrelation coefficient curves (middle row), and PE curves (bottom row) of the output of the DM laser subjected to optical feedback. The left, middle, and right columns are for the feedback ratios of −14.5 dB, −10.5 dB, and −1.5 dB, respectively. In Figure 2A, the red line represents the DM laser’s time trace without optical feedback. From the time traces in Figure 2, it can be seen that the laser exhibits random fluctuations in all three feedback ratios, indicating chaos dynamics. To identify the time-delay signatures, their corresponding autocorrelation coefficient C, as a function of the delay time, is calculated and shown in the middle row of Figure 2. At a feedback ratio of −14.6 dB (Figure 2D), a significant peak at approximately 116.8 ns, corresponding to the feedback round trip time (τ_1_), is observed. This peak, referred to as a time-delay signature, is quantified using the peak value of the autocorrelation coefficient at around the feedback round trip time (C_p_). In Figure 2D, the time-delay signature is approximately 0.82. As the feedback ratio increases to −10.5 dB, the time-delay signature decreases to approximately 0.39, as shown in Figure 2E. Further increasing the feedback ratio to −1.5 dB results in a reduced time-delay signature of approximately 0.24, as shown in Figure 2F.

We also utilize PE to investigate the time-delay signature, as shown in the bottom row of Figure 2. Figures 2G–I show many troughs that are attributable to harmonics and sub-harmonics of the feedback round trip time. Notably, the deepest troughs, occurring at approximately τ_1_ ≈ 116.8 ns, are less pronounced in the PE analysis than the autocorrelation coefficient analysis (middle row of Figure 2). Therefore, we focus on the autocorrelation coefficient for the remaining investigation.

**FIGURE 3:**
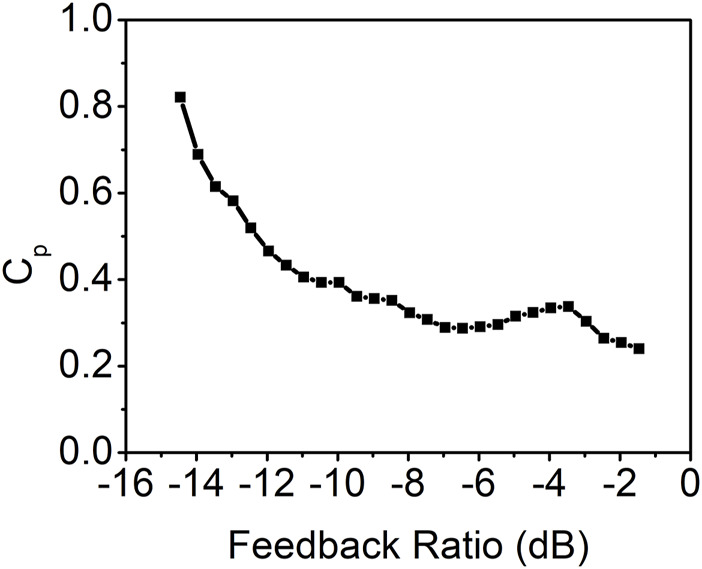
Time-delay signature as a function of the feedback ratio for the conventional feedback.

The peak value of the autocorrelation coefficient at the feedback round trip time as a function of the feedback ratio is calculated and presented in Figure 3. The result indicates that the time-delay signature decreases as the feedback ratio increases when the feedback ratio is less than approximately −7 dB. Beyond this threshold, the time-delay signature begins to rise as the feedback ratio increases, peaking around a feedback ratio of −3.5 dB. Subsequently, with further increases in the feedback ratio, the time-delay signature diminishes once more. Notably, the minimum time-delay signature of 0.24 is achieved at the maximum feedback ratio of −1.5 dB. This is corroborated by the autocorrelation coefficient curve displayed in Figure 2F, which distinctly identifies the time-delay signature at 116.8 ns.

**FIGURE 4:**
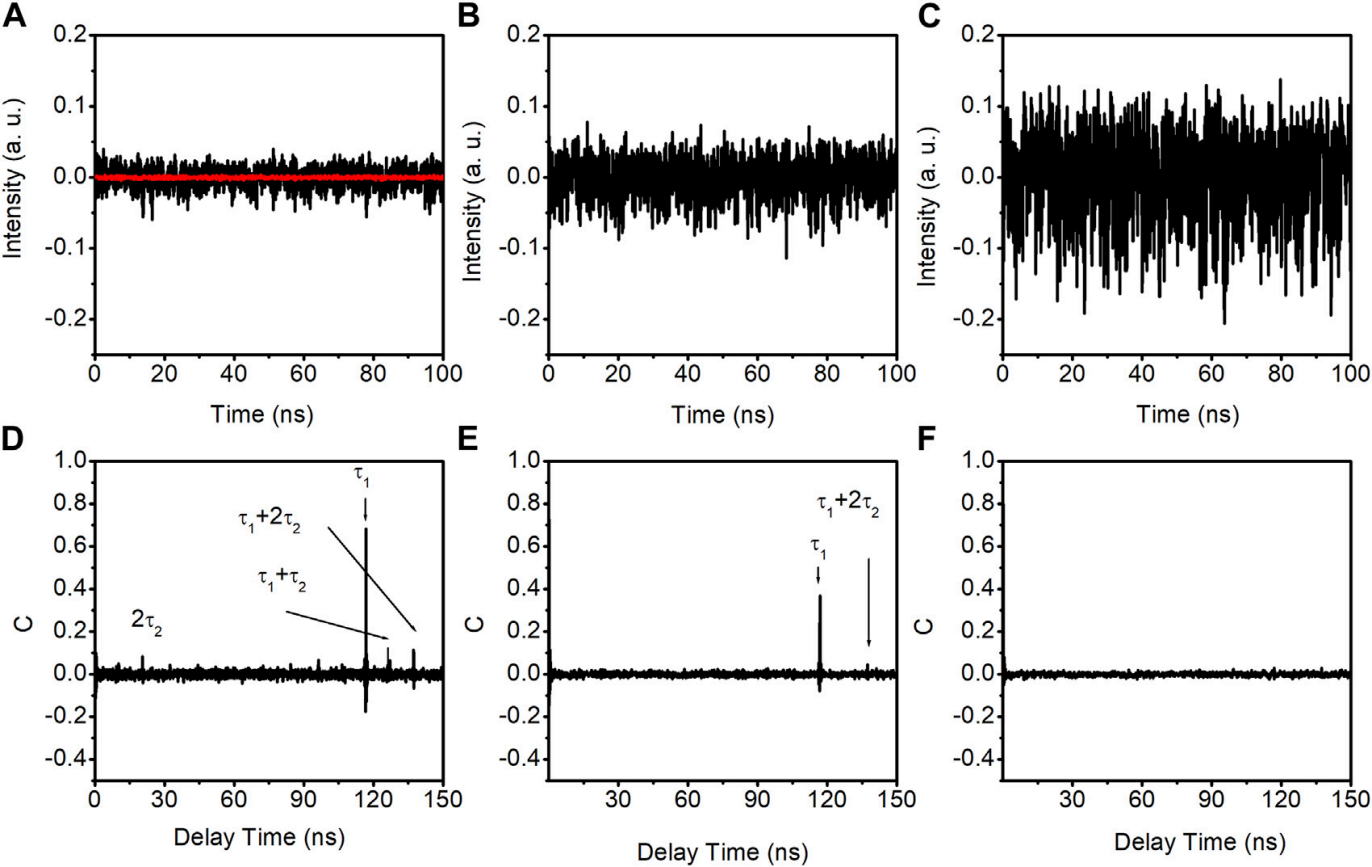
Time traces, ACC curves and PE curves of the output of the DM laser with photonic filter feedback with the coupling ratio of 50%. **(A–C)** are time traces with the feedback ratios of −14.5 dB, −10.5 dB, and −1.5 dB, respectively. **(D–F)** are autocorrelation coefficient curves with the feedback ratios of −14.5 dB, −10.5 dB, and −1.5 dB, respectively. The red line in **(A)** is the free-running DM laser output.

Moving to photonic filter feedback, we connect ports 2 and 4 of the VFC. Initially, the coupling ratio is set at 50%, equally splitting the powers between ports 3 and 4. Figure 4, shows the time traces (upper row) and autocorrelation coefficient curves (bottom row) for the DM laser with photonic filter feedback. The feedback ratios for the left, middle, and right columns in Figure 4 match those in Figure 2: −14.5 dB, −10.5 dB, and −1.5 dB, respectively. The red line in Figure 4A corresponds to the free-running DM laser’s output.

Similar to conventional feedback, the laser exhibits random fluctuations in all three feedback ratios, indicative of chaotic dynamics. The corresponding autocorrelation coefficient curves are displayed in the bottom row of Figure 4. It can be seen in Figure 4D that aside from the highest peak at approximately 116.8 ns, smaller peaks appear at approximately 20.5 ns, 127.05 ns, and 137.3 ns. These additional peaks are attributed to the time delay introduced by the ring cavity recirculation. Each recirculation within the ring cavity introduces a delay time (τ_2_) of approximately 10.25 ns. The highest peak has a value of approximately 0.68. In the case of −10.5 dB feedback ratio, as shown in Figure 4E, the maximum peak value decreases to approximately 0.37. When the feedback ratio increases to approximately −1.5 dB, as demonstrated in Figure 4F, no distinguishable peaks are observed. The time-delay signature has been completely concealed.

**FIGURE 5:**
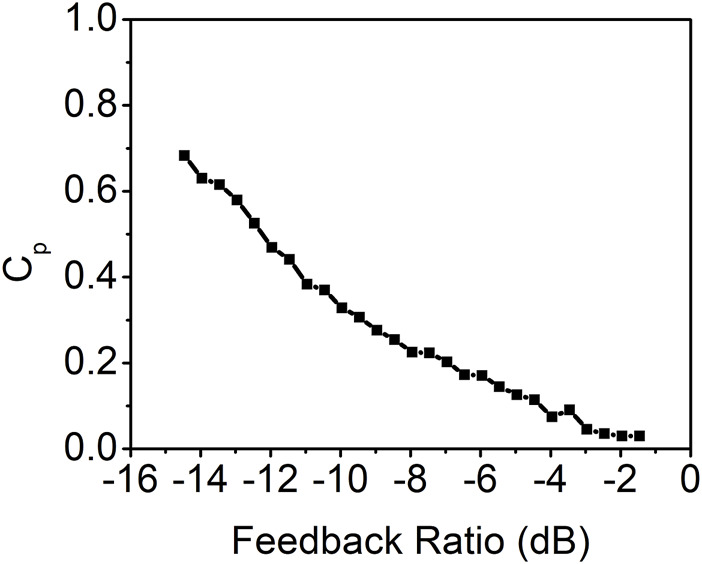
Time-delay signature as a function of the feedback ratio for the photonic filter feedback with the coupling ratio of 50%.

The maximum peak value of the autocorrelation coefficient at the feedback round trip times (τ_1_, τ_1_+τ_2,_ τ_1_+2τ_2_, 2τ_2_, or other combinations) as a function of the feedback ratio is presented in Figure 5. The result demonstrates a consistent decrease in the time-delay signature as the feedback ratio increases. When the feedback ratio reaches approximately −2.0 dB, the time-delay signature value is approximately 0.03. Further increases in the feedback ratio yield minimal changes in the time-delay signature due to the absence of distinguishable peaks in the autocorrelation curves.

**FIGURE 6:**
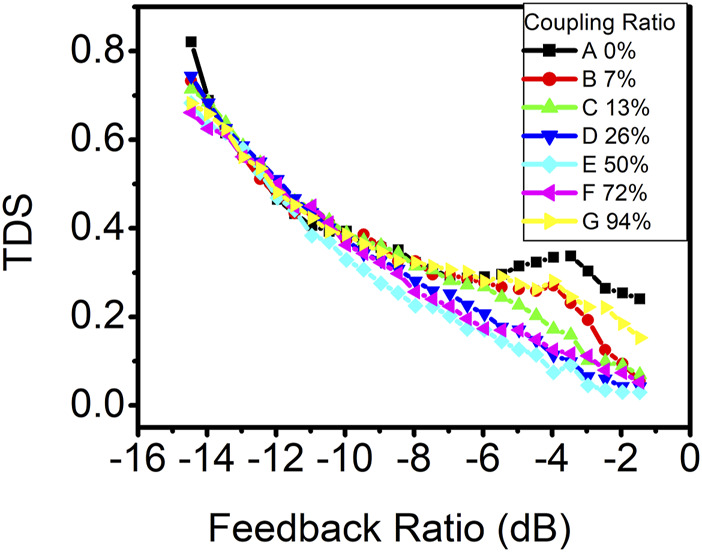
Time-delay signature as a function of the feedback ratio in the DM laser with optical feedback. Curve A represents conventional optical feedback. Curves B, C, D, E, F, and G represent photonic filter feedback with the coupling ratio of 7%, 13%, 26%, 50%, 72%, and 94%, respectively.

The influence of the coupling ratio of the photonic filter on the time-delay signature is also explored. In Figure 6, curve A represents the scenario with conventional feedback, while the remaining curves correspond to setups involving photonic optical feedback, each with different coupling ratios. It is evident that at lower feedback ratios, photonic filter feedback does not show any advantage in suppressing the time-delay signature compared to conventional feedback. However, as the optical feedback intensity increases, the addition of photonic filter feedback proves advantageous in suppressing the time-delay signature, particularly when the coupling ratio approaches 50%.

### 4.2 VCSELs

**FIGURE 7:**
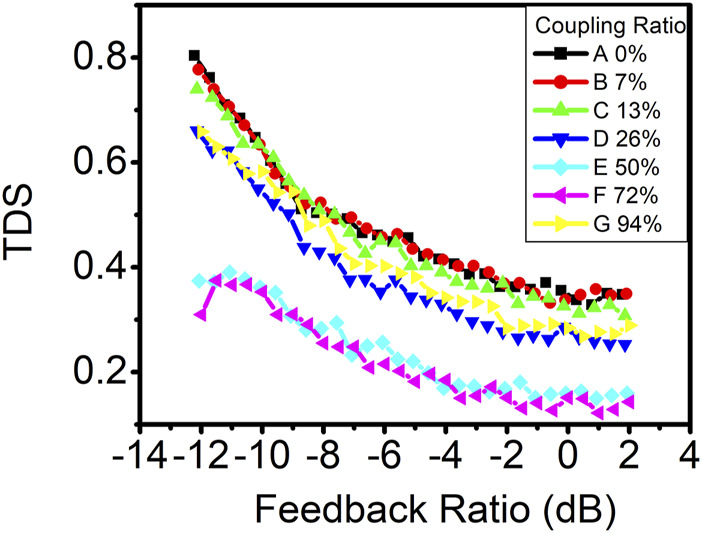
Time-delay signature as a function of the feedback ratio in VCSEL with various coupling ratios.

To investigate whether the concealment of the time-delay signature is solely attributable to photonic filter feedback, we conducted a similar experiment using VCSEL. The threshold current of VCSEL used in this experiment is 1.8 mA at the room temperature and is biased at 4 mA. Figure 7 displays the time-delay signature as a function of the feedback ratio in VCSEL with various coupling ratios. Notably, the addition of photonic filter feedback at coupling ratios of 50% or 72% effectively suppressed the time-delay signature across all feedback ratios, which is similar to the results observed in DFB lasers (Cui et al., 2022). However, for coupling ratios below 13%, the time-delay signature exhibits little deviation from conventional optical feedback, in contrast to DM lasers, where time-delay signature suppression with a photonic filter feedback is primarily observed at higher feedback ratios. Remarkably, even a lower coupling ratio of 7% still significantly contributes to time-delay signature suppression in DM lasers at higher feedback ratios. The optimal coupling ratio for time-delay signature suppression in VCSEL is determined to be 72%. The minimum time-delay signature achieved in VCSEL is approximately 0.12 at a feedback ratio of approximately 1.0 dB with the coupling ratio of 72%, which is higher than the minimum time-delay signature of 0.03 observed in the DM laser.

**FIGURE 8:**
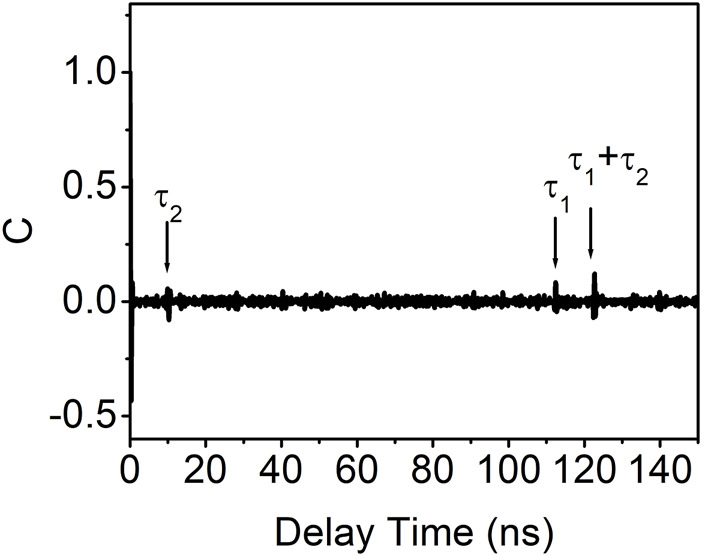
Autocorrelation coefficient curve of the output of VCSEL with photonic filter feedback with the coupling ratio of 72% and the feedback ratio of 1.0 dB.

Figure 8 shows the autocorrelation coefficient curve obtained under the influence of photonic filter feedback with an optimal coupling ratio and optical feedback ratio in VCSEL. This curve exhibits three distinct peaks, with delay times of approximately 10.25 ns, 112.45 ns, and 122.7 ns, corresponding to τ_2_, τ_1_, and τ_1_+τ_2_, respectively. This observation indicates that the presence of photonic filter feedback in VCSEL is unable to entirely eliminate the time-delay signature.

## 5 Conclusion

In the context of physiological systems, which exhibit complex nonlinear dynamics and self-organized pattern formation, the study of analogous dynamics in semiconductor lasers subjected to delayed optical feedback provides valuable insights. Building on the pioneering work and the extensive literature on the subject, lasers have served as a paradigm for understanding complex nonlinear dynamics in biological and physiological systems. In this study, we conducted experimental investigations into the time-delay signature of semiconductor lasers under conventional feedback and photonic filter feedback conditions. Specifically, two types of semiconductor lasers, namely, DM lasers and VCSELs, are comprehensively examined. Our findings highlight the substantial advantages of photonic filter feedback in time-delay signature suppression, particularly evident in the case of DM lasers. At the optimal coupling and optical feedback ratios, we achieved remarkable time-delay signature reduction, with the time-delay signature minimized to as low as 0.03, effectively concealed within the background noise. For DM lasers, the benefits of photonic filter feedback in time-delay signature suppression manifest primarily at higher feedback ratios. Conversely, in the case of VCSELs, photonic filter feedback proves advantageous across a wider spectrum of feedback ratios, particularly in the coupling ratio range of approximately 50%–72%. Nevertheless, it is worth noting that in VCSELs, while photonic filter feedback induces significant time-delay signature suppression with an appropriate coupling ratio, a residual time-delay signature remains discernible. The reason the photonic filter can suppress the time-delay signature is that the photonic filter feedback is equivalent to optical feedback from multiple external cavities with different lengths, and due to the multiple Vernier effect, the time-delay signature is suppressed. The disparity between DM lasers and VCSELs can be attributed to their respective laser structures. DM lasers feature multiple etching features along the ridge waveguide, which alter the characteristics of the laser spectrum. This modification, in turn, mitigates the occurrence of recurring features induced by an optical feedback. Combining the multiple Vernier effect with the modified laser spectrum totally conceals the time-delay signature in the discrete-mode laser with photonic filter feedback. However, the effectiveness of photonic filter feedback for time-delay signature suppression is diminished in VCSELs because they lack the same special spectrum characteristics as discrete-mode lasers. Without the specific spectrum provided by the multiple etching features along the ridge waveguide, the photonic filter cannot totally suppress the time-delay signature in VCSELs.

This research serves not only to enhance our comprehension of time-delay signature control in semiconductor lasers but also holds significance in the context of physiological phenomena. Given the parallels between semiconductor laser dynamics and physiological processes marked by time-delay effects, the insights gained from this study can contribute to the better understanding and management of physiological phenomena, opening new avenues for research and applications in the realm of controlling and regulating complex physiological systems.

## Data Availability

The raw data supporting the conclusion of this article will be made available by the authors without undue reservation.
